# Exploring the Relationship Between CAIDE Dementia Risk and EEG Signal Activity in a Healthy Population

**DOI:** 10.3390/brainsci14111120

**Published:** 2024-11-04

**Authors:** Alice Rodrigues Manuel, Pedro Ribeiro, Gabriel Silva, Pedro Miguel Rodrigues, Maria Vânia Silva Nunes

**Affiliations:** 1 Faculdade de Ciências da Saúde e Enfermagem, Universidade Católica Portuguesa, Rua Palma de Cima, 1649-023 Lisboa, Portugal; s-alromanuel@ucp.pt (A.R.M.); mnunes@ucp.pt (M.V.S.N.); 2 CBQF—Centro de Biotecnologia e Química Fina—Laboratório Associado, Escola Superior de Biotecnologia, Universidade Católica Portuguesa, Rua Diogo Botelho 1327, 4169-005 Porto, Portugal; s-pmsbribeiro@ucp.pt (P.R.); g.arsilva@hotmail.com (G.S.); 3 Centro de Investigação Interdisciplinar em Saúde (CIIS), Faculdade de Ciências da Saúde e Enfermagem, Universidade Católica Portuguesa, Rua Palma de Cima, 1649-023 Lisboa, Portugal

**Keywords:** CAIDE, aging, risk of dementia, electroencephalogram, deceleration effect, correlation analysis

## Abstract

Background: Accounting for dementia risk factors is essential in identifying people who would benefit most from intervention programs. The CAIDE dementia risk score is commonly applied, but its link to brain function remains unclear. This study aims to determine whether the variation in this score is associated with neurophysiological changes and cognitive measures in normative individuals. Methods: The sample comprised 38 participants aged between 54 and 79 (M = 67.05; SD = 6.02). Data were collected using paper-and-pencil tests and electroencephalogram (EEG) recordings in the resting state, channels FP1 and FP2. The EEG signals were analyzed using Power Spectral Density (PSD)-based features. Results: The CAIDE score is positively correlated with the relative power activation of the θ band and negatively correlated with the MMSE cognitive test score, and MMSE variations align with those found in distributions of EEG-extracted PSD-based features. Conclusions: The findings suggest that CAIDE scores can identify individuals without noticeable cognitive changes who already exhibit brain activity similar to that seen in people with dementia. They also contribute to the convergent validity between CAIDE and the risk of cognitive decline. This underscores the importance of early monitoring of these factors to reduce the incidence of dementia.

## 1. Introduction

According to the Regional Information Center for Western Europe, “Globally, the number of people aged 80 and over is expected to triple by 2050, from 137 million in 2017 to 425 million in 2050” [1]. However, while there is an increase in average life expectancy, it may not necessarily translate to healthy years of life, as there is a rise in age-related diseases and disabilities, such as dementia [2].

Thus, the increase in older adults alludes to physiological and pathological aging, a rather complex and challenging area. What, then, delineates the boundary between these two aging processes?

### 1.1. Normative vs. Pathological Aging

While there is no consensus regarding the concept of “Normative Aging,” it can be characterized by the behavioral changes that become apparent with advancing age, resulting from alterations in the nervous system not caused by neurological injury [3]. These transformations associated with physiological aging primarily occur at the level of the prefrontal cortex [4] and the hippocampus [5], although with less significance in the latter. No single process can elucidate or clarify all the changes that occur during aging, as it is an extraordinarily complex process that varies significantly from person to person [6]. Cognitive activity is related to brain function and, subsequently, to structural and physiological changes, declining heterogeneously with advancing age [7]. Pathological changes begin decades before cognitive symptoms become evident, starting with synaptic loss and progressing to significant brain atrophy [8].

According to Park (2000) [9], language, visuospatial functions, and semantic memory generally remain preserved, while spontaneous memory, attention, and executive function slightly decline. In this regard, it is the progressive cognitive and behavioral decline resulting from brain aging that assumes greater prominence, constituting the primary symptom of a group of syndromes known as dementia [10]—the most common cause of disability [2].

Regarding its evolution, since 2007, there has been a growing interest in studying the preclinical stage of dementia, thus giving rise to the concept of the prodromal phase [11]. This phase represents the initial period of the disease, during which the difficulties presented are indistinguishable from normal cognitive aging due to a long preclinical stage and the existence of an intermediate stage known as mild cognitive impairment (MCI), which may manifest specific cognitive difficulties with a mild degree of impairment. Typically, dementia biomarkers are already evident [12]. It is worth noting that not all individuals experiencing this phase will necessarily develop dementia in the future, thus emphasizing the importance of understanding the risk factors that may or may not influence this progression. Clinical stages can range from cognitively normal to mild cognitive impairment and dementia, highlighting a continuum that extends over years.

More recent data indicate that, based on the percentage of controlled risk factors, it is theoretically possible to delay or prevent one-third of dementia cases [13]. In other words, even after the onset of the disease, lifestyle modification can reduce the rate of cognitive decline [14]. Thus, despite the increase in life expectancy, the incidence of dementia has decreased in many countries. This reversal in the expected pattern underscores the importance of deepening our understanding of the impact of these factors by calculating a global level of associated risk and potential physiological changes [15].

A dementia risk factor is a characteristic, condition, or behavior that increases the likelihood of an individual developing dementia. However, despite their potential influence, it is not precisely known how risk factors may cause it, thus necessitating an understanding of the underlying mechanisms of dementia. Consequently, by intervening in the risk factors, it is hoped that the course of the disease can be modified to delay or reduce its onset [13,16,17].

### 1.2. Dementia Risk Assessment

In addition to simple cognitive assessments (e.g., MMSE), risk scores (e.g., LIBRA, CAIDE) are often used to identify at-risk individuals. Given the multifactorial nature of dementia, identifying individuals at higher risk of developing dementia based on a single risk factor becomes insufficient. Therefore, opting for an estimation of dementia risk using multifactorial risk scores is advantageous for recognizing individuals who may benefit most from risk reduction strategies [18].

A risk score is a weighted combination of risk factors that reflects the probability of developing a disease, condition, or disorder. Typically, an additive approach is adopted, where the weighting of different individual risk factors is summed up to obtain a total value. This weighting is generally established based on analyses of cohort studies or meta-analyses [19]. Multifactorial risk scores may include both non-modifiable factors (e.g., genetics, age) as well as modifiable factors (e.g., physical activity, cholesterol) [18].

The Cardiovascular Risk Factors, Aging, and Dementia (CAIDE) Dementia Risk Score, validated by Kivipelto and colleagues (2006) [20], reflects an estimate of dementia risk over 20 years based on an individual’s midlife risk factor profile. It was developed in a Finnish population cohort aged 39 to 64 years, considering demographic and biometric data and including easily measurable factors such as age, sex, education level, body mass index (BMI), systolic blood pressure, total cholesterol, and physical activity [21]. Its validity has subsequently been supported by studies seeking to understand the risk detected by CAIDE and its correlation with neurological bases. In 2015, Vuorinen and colleagues [22], through a population-based study utilizing magnetic resonance imaging, demonstrated that a higher CAIDE dementia risk score in midlife is associated with changes in white matter and medial temporal lobe atrophy. Additionally, a higher CAIDE dementia risk score tends to identify patients with a greater likelihood of having cerebral pathology (e.g., neurodegeneration, beta-amyloid (Aβ) deposition, and cerebrovascular changes).

Consistently, in 2017, Stephen and colleagues [23] aimed to associate high CAIDE scores not only with white matter lesions, decreased gray matter, reduced hippocampal volume, and decreased density in areas commonly associated with Alzheimer’s disease but also with medial temporal lobe atrophy in the presence of increased apolipoprotein. However, the literature in this area is limited, lacking references to metabolic and electrophysiological functioning, underscoring the importance and necessity of further research.

### 1.3. Electroencephalogram and Deceleration Effect

The electroencephalogram (EEG) is a recording technique that reflects changes in the brain’s bio-electrical activity, which can be used to recognize and monitor various diseases [24]. Electroencephalography (EEG) signals are categorized into five conventional frequency bands: delta (δ, 0.5–4 Hz), which is dominant during deep sleep and associated with unconscious states; theta (θ, 4–8 Hz), observed during light sleep, drowsiness, or meditation and linked to creativity and intuition; alpha (α, 8–13 Hz), present during relaxed, wakeful states with closed eyes, indicating relaxation and calmness; beta (β, 13–30 Hz), seen during active thinking, problem-solving, and focus, associated with alertness and active concentration; and gamma (γ, 30–40 Hz), related to high-level cognitive functioning, information processing, and perception [25].

EEG is recognized as a physiological biomarker that allows for detecting dementia in early stages through the analysis and processing of EEG signals, which reflect neuronal activity. These data, combined with neuropsychological tests, aid in the comprehensive characterization of the dementia progression spectrum before significant cognitive decline. The implications of dementia on EEG can be summarized as a slowing effect on brain waves [26,27,28].

According to Doan et al. (2021) [29], numerous studies have aimed to demonstrate that EEG biomarkers at rest and components of evoked potentials (ERPs) obtained from EEG signals are reliable in distinguishing dementia from normal controls and other neurological disorders. The authors showed that, in a sample of 122 participants (including 35 with dementia and 87 control individuals), measurements in the resting-state EEG in the frontal lobe revealed a shift from high-frequency bands (i.e., α and β) to low-frequency bands (i.e., δ and θ) in individuals with dementia.

Similarly, Rodrigues and Teixeira (2011) [30], after analyzing the spontaneous EEG activity of 20 patients diagnosed with AD and 14 control subjects, demonstrated the contribution of EEG as a complementary diagnostic examination through the slowing effect in the captured electrical signals, allowing for a more accurate and early diagnosis of AD. The study involved EEG recordings made in individuals in a relaxed state with their eyes closed, aiming to obtain the highest amount of artifact-free EEG data. In individuals with AD, there was an increase in relative power in the δ (*p* < 0.01) and θ frequencies (*p* < 0.0005), while a decrease occurred in the α (*p* < 0.05), low-β (*p* < 0.001), and high-β frequencies (*p* < 0.005), compared to individuals in the control group. The most significant differences were observed in the θ and β bands.

Given this, the slowing effect in dementia, characterized by decreased activity from high-frequency to low-frequency bands, corresponds to a progressive reduction in inter-neuronal connections and information transmission and the subsequent slowing of oscillatory activity (spectral deceleration). This effect is present even years before the onset of clinical symptoms—the prodromal phase [31]. However, little to nothing is known about its relationship with risk factors, especially in the absence of cognitive decline. In 2020, Rossini et al. conducted a literature review to assess EEG’s capability to detect signs indicative of individuals’ dementia risk, with the goal of early intervention in modifiable risk factors and pharmacological intervention. Interest in neurophysiological biomarkers has emerged alongside understanding their advantages, such as instruments like EEG.

### 1.4. Research Questions and Study Objectives

Currently, there exists a significant gap in scientific knowledge regarding the association between the CAIDE dementia risk score and the underlying neurophysiological changes that occur before the manifestation of dementia. Understanding these changes may provide insights into the underlying disease mechanisms, enabling more precise and timely interventions.

Therefore, this study was conducted with the following main objectives:

- Assess traditional frequency band power distributions to characterize the brain electrical signals of the participants via Power Spectral Analysis of electroencephalographic signals.
- Investigate the relationship between variations in dementia risk scores, as assessed by CAIDE, and the characteristics of brain electrical signals recorded by EEG and cognitive measures in normative individuals.
- Explore whether a deceleration effect in the spectrum, similar to that observed in dementia, appears in individuals with high dementia risk scores before the onset of cognitive decline.

## 2. Methodology

This section describes each step of the experimental study, as well as the introduction of the built database.

**Figure 1:**
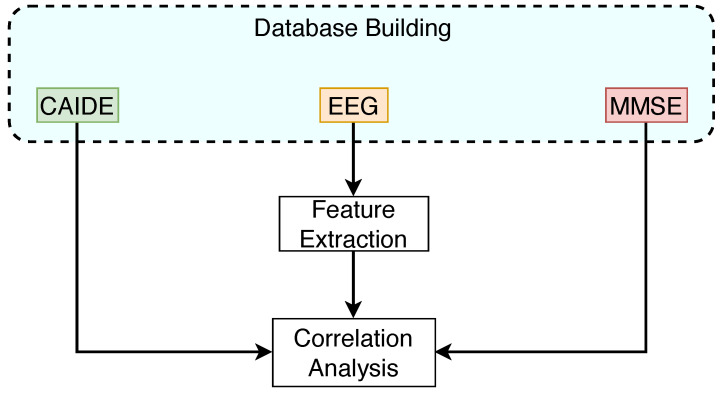
Methodology workflow diagram.

Figure 1 illustrates the Methodology Diagram for the steps described below.

### 2.1. Ethical Considerations

This research project followed the principles established in the World Medical Association’s Declaration of Helsinki [32], ensuring adherence to all requisite ethical procedures for protecting and respecting research participants (Favorable Decision from the Health Ethics Committee, issued in February 2023, by the Portuguese Catholic University—Project number 236). It should be noted that all participants gave their consent before participating in this study.

### 2.2. The Database

The following inclusion criteria were considered: participants had to (a) ensure the necessary conditions for the execution of neuropsychological tests, showing no sensory, motor, or visuoperceptive difficulties or impairments, (b) have Portuguese as their native language, and (c) voluntarily agree to participate in the study.

Additionally, participants were excluded if they met the following exclusion criteria: (a) diagnosed with dementia, (b) presented neurological conditions affecting the EEG signal, (c) obtained scores below the expected average for the population on the MMSE test, (d) scored severe depression on the BDI-II, or (e) were illiterate.

The database is composed of 47 participants. However, the data for 9 participants were discarded due to errors in the electroencephalographic signal capture. Thus, the sample in the present study consists of 38 participants, aged between 54 and 79 years (M = 67.05, SD = 6.02), with 23 females (60.5%) and 15 males (39.5%). Additionally, 71.1% of the participants had completed primary education or less, with six or fewer years of formal schooling. See Table 1 for a more detailed characterization of the sample.

### 2.3. Measures

#### 2.3.1. Sociodemographic Data Questionnaire

Each participant completed a pencil-and-paper questionnaire comprising questions related to their sociodemographic characteristics, such as gender, age, education level, and physical activity.

#### 2.3.2. Mini-Mental State Examination (MMSE)

This global cognitive screening instrument, developed in 1975 by Folstein and colleagues [33], assesses domains of temporal and spatial orientation, attention, language, writing, calculation, immediate and delayed memory, and visuoconstructional ability (cube drawing). It is the most commonly used instrument in research for global cognitive assessment [34]. The administration time of the test varies between 5 and 10 min. In this study, the version validated for the Portuguese population by Guerreiro and colleagues (1994) [35] was used to assess the participants’ mental status, using it as an exclusion criterion. Scores range from 0 to 30, with higher scores indicating better cognitive status. Good psychometric properties of internal consistency (Cronbach’s alpha = 0.856) were reported. The following cutoff points were used for cognitive impairment: one to eleven years of education: impairment ≤ 22 points; twelve years of schooling: impairment ≤ 27 points.

#### 2.3.3. Beck Depression Inventory (BDI-II)

This is an updated version of Beck and colleagues’ (1996) [36] questionnaire for the self-assessment of depression, in which participants must report on the presence of specific symptoms over the past two weeks. It assesses 21 domains that, together, reflect an overall score of depressive symptomatology: sadness, pessimism, past failures, loss of pleasure (anhedonia), feelings of guilt, feelings of punishment, self-contempt, self-criticism, suicidal thoughts or wishes, crying, agitation, loss of interest, indecision, self-depreciation, loss of energy, changes in sleep habits, instability, changes in appetite, difficulties concentrating, tiredness or fatigue, and loss of sexual interest. The questionnaire has an administration time of 5 to 10 min and a total score of 63 points, with a higher score indicating greater severity of depressive symptomatology. In this study, the version adapted for the Portuguese population by Campos and Gonçalves (2011) [37] was used, which demonstrated good values of internal consistency in two different samples, with Cronbach’s alphas of 0.90 and 0.91, respectively. The data can be interpreted as follows: mild symptomatology (≤13 points), mild depression (14–19 points), moderate depression (20–28 points), and severe depression (29–63 points).

#### 2.3.4. CAIDE Dementia Risk Score

This scale allows the calculation of a total score of risk factors, including easily measurable factors such as age, education, blood pressure, body mass index (BMI), cholesterol, and physical activity, with its validity and specificity in predicting dementia verified: see Table 2. In this research, an adapted version of the scale by Stephen and colleagues (2021) [18] was used to infer the level of dementia risk in individuals. The higher the score obtained, the higher the risk of dementia, which can range from 0 to 15 points.

### 2.4. Procedure

The data were collected at a senior institution in a rural setting. Information about the study was disseminated through an oral presentation at the collection site. Informed consent (see Supplementary Material S1) was obtained in written form from all participants, which included a brief explanation of the study, assurance of confidentiality, and the option to withdraw at any time. In the first stage (lasting 30 min), neuropsychological tests (i.e., MMSE, BDI-II) and the Sociodemographic Data Questionnaire (see Supplementary Material S1) were administered for participant inclusion/exclusion. In the second stage (also lasting 30 min), conducted one week after the first assessment, electroencephalographic (EEG) recordings (resting-state EEG) were collected. The dementia risk score was obtained using CAIDE criteria. The institution provided blood pressure, BMI, and cholesterol data upon participant consent. The two data collection stages were scheduled based on the respective dates of annually performed routine analyses (all values correspond to data collected up to two months before the assessment). The time interval between the first and second assessment stages was one week.

### 2.5. EEG Collection Procedure

The electrodes were positioned according to the International 10–20 System, categorized with even numbers if they were on the right side of the brain and odd numbers on the left side (e.g., FP1 electrode in the left prefrontal area, closer to the interhemispheric fissure), separated from each other by distances of 10% or 20% between the nasion (point between the forehead and the nose), inion (most prominent point of the occipital part of the brain), and preauricular regions.

The present investigation collected EEG signals while resting, with participants lying down with closed eyes. After conducting a pre-test (where the number of electrodes and the signal sampling frequency—Hertz—were varied), the most advantageous procedure for the study objectives was determined. Thus, 15 continuous minutes of resting-state activity was recorded at a frequency of 200Hz using two recording channels, FP1 and FP2, and one reference channel (without brain activity)—the right ear. Two electrodes were applied to each frontal channel, with only one required for the reference channel. Therefore, the assembly scheme involved placing the five electrodes on the individual’s scalp—according to the International 10–20 System—to acquire the electrical impulses, which were subsequently recorded, digitized, and amplified.

For signal recording and acquisition, the OpenSignals software version 2.2.5 was utilized. An initial effort was required to ensure accuracy in electrode placement, aiming to prevent resistance to the passage of electric current, which would lead to unavoidable noise and unsatisfactory results. Participants who had less than four hours of sleep the night before the data collection session were excluded.

As mentioned, transformations associated with normative aging primarily affect the prefrontal cortex, a crucial area for maintaining higher cognitive functions [4]. It is also known that dementia risk factors may trigger neurophysiological changes similar to those of typical aging, accelerating this process. On the other hand, the literature indicates that higher CAIDE dementia risk scores are associated with a greater risk of developing brain pathology [22].

It is essential to highlight that the frontal lobe is susceptible to the onset of age-related changes. As mentioned above, frontal regions are presumed to be the first to be affected phylogenetically and ontogenetically by brain aging [38]. In addition, the frontal electrodes of the EEG record not only frontal regions but also nearby areas in the temporal lobe, enabling the early detection of potential information loss, which may be indicative of dementia. The analysis of the prefrontal regions (FP1 and FP2) showed that these regions are sensitive to dementia-related changes and physiological aging, indicating potential pathological manifestations.

In summary, choosing these regions optimizes the detection process, allowing for the measurement of activity in the frontal and temporal areas, reflecting the complexity of brain changes. The signal propagation effect underscores the importance of paying attention to identifying information losses [39], making this approach crucial for the early detection of dementia risk effects and brain health monitoring.

### 2.6. EEG Data Processing

MATLAB 2023b software was employed to develop this task.

#### 2.6.1. EEG Preprocessing

**Figure 2:**
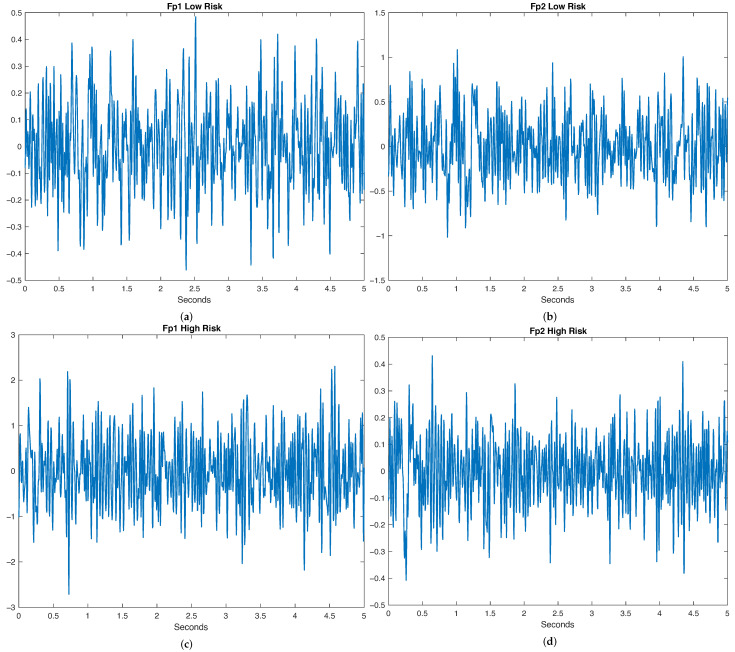
EEG signals for the FP1 and FP2 channels for both the Low- and High-Risk classes. (**a**) FP1 Low Risk, (**b**) FP2 Low Risk, (**c**) FP1 High Risk, (**d**) FP2 High Risk.

The EEG signals were denoised using the wICA (Wavelet + Independent Component Analysis) tool [40] for removing electrooculographic (EOG) and muscle artifacts. Starting with ICA decomposition via FastICA, we applied wavelet thresholding to the de-mixed independent components, not the observed EEG. Using the Daubechies 6 mother wavelet, we performed a discrete wavelet transform on these components. We used universal fixed-form thresholding proposed by Coifman and Donoho (1995) [41] for EEG denoising, which is based on the noise level and the number of data points. Finally, we transformed the signal back into the sensor space to obtain the denoised EEG, and we digitally filtered its spectrum by using an Elliptic filter of the 16th order with 1 and 40 Hz cutoff frequencies and a Stop attenuation band of 60 dB, and then the mean value was removed. Examples of FP1 and FP2 channels’ filtered signals for low-risk and high-risk classes are shown in Figure 2.

#### 2.6.2. EEG Time–Frequency Analysis Through Power Spectral Density and Feature Extraction

A common approach to analyzing the spectrum of EEG signals involves studying their frequency components [42]. The Fourier Transform (FT) is frequently employed to extract spectral information from stationary signals. The coefficients of this transform result from computing a series of inner products between the signal and sinusoidal functions of infinite duration.

For an EEG signal x(t) sampled at a frequency $f_{s}$, where $f_{s}$ is the inverse of the sampling period, a discrete signal is obtained as $x\left[n\right]\;=\;x\left(n\cdot T_{s}\right)$, with n=0,…,N−1, where *N* represents the number of samples. Applying the Discrete-Time Fourier Transform (DTFT) to this signal yields the spectrum X[k], where k=0,…,N−1. It is assumed that the input signal x[n] is periodic with a fundamental period *N* and a fundamental frequency Ω=2π/N. The variable *k* corresponds to the frequency of the sinusoid associated with X[k], and the discrete frequency is given by $\Omega =k\Omega_{0}$. The following formulas illustrate the DTFT analysis associated with the signal.

(1) $$x\left[n\right]\;=\;\frac{1}{N}\sum_{k=0}^{N-1}X\left[k\right]\cdot e^{j\cdot k\cdot \Omega_{0}\cdot n},\;n\;=\;0,\ldots ,N-1$$

(2) $$X\left[k\right]\;=\;\frac{1}{N}\sum_{n=0}^{N-1}x\left[n\right]\cdot e^{-j\cdot k\cdot \Omega \cdot n},\;k\;=\;0,\ldots ,N-1$$

According to these equations, the coefficients of the signal x[n] are represented by X[k]. Both form a DTFT pair, allowing the recovery of x[n] from the *N* values of X[k], and vice versa. Both x[n] and X[k] provide a complete description of the signal [43,44].

One way to analyze signal characteristics is to observe the power distribution in the frequency domain. The Power Spectral Density (PSD) function is commonly used for this purpose [45], assuming the analyzed signal is a power signal. A typical method to estimate the PSD is by calculating it as the DTFT of the autocorrelation function of the EEG signal (Wiener–Khinchin theorem), as both are a Fourier Transform pair [45]. The following equation illustrates an estimate of the autocorrelation function $R_{xx}\left[u\right]$ from the discrete signal x[n]:

(3) $$R_{xx}\left[u\right]\;=\;\left\{\begin{array}{cc} \frac{1}{N}\sum_{n=0}^{N-m-1}x\left[n\right]\cdot x\left[n+u\right], & \mathrm{if}\;u\geq 0\\ R_{xx}^{*}\left[-u\right], & \mathrm{if}\;u<0 \end{array}\right.$$

The subsequent equation represents the estimation of the PSD, PSD[k], where DTFT{x} denotes the Discrete Fourier Transform [45,46]:

(4) $$PSD\left[k\right]\;=\;\frac{1}{N}\mathrm{DTFT}\left\{R_{xx}\left[u\right]\right\}\;=\;\frac{1}{N}\sum_{u=0}^{N-1}R_{xx}\left[u\right]\cdot e^{-j\frac{2\cdot \pi k}{N-1}},k\;=\;0,\ldots ,N-1$$

To simplify the computation of some features from the PSD, the PSD was normalized between 0 and 1:

(5) $$PSD_{n}\left[k\right]=\frac{PSD[k]}{\sum_{k=0}^{N_{T}-1}PSD\left[k\right]}$$

(6) $$PSD_{n}\left(f\right)=PSD_{n}\left[\frac{k*f_{s}}{N_{T}}\right]$$

where $N_{T}$ is the length of the $PSD_{n}$ and *f* the frequency bins.

**Figure 3:**
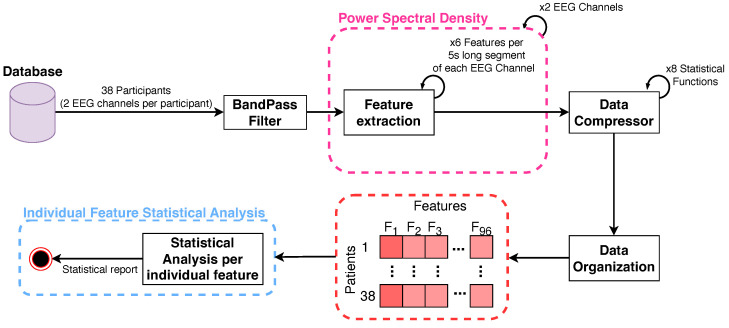
EEG processing workflow diagram.

Six features (check Table 3 for more information) were collected from each signal $PSD_{n}$ computed using a time-series non-overlapping sliding windowing processing analysis of 1 s length from a total signal length of 5 min. This means that for each 1 s segment of the signal resulting from the windowing analysis, a $PSD_{n}$ was estimated by using Equation (6) to collect features over time. Then, the resulting time series per feature over time comprised eight distinct statistical functions: maximum (max), minimum (min), mean, median, mode, standard deviation (SD), Variance (var), and Covariance (cov) [47]. At the end of the process, the data matrix, comprising all 5-min time-series vectors of features extracted from both signals for all patients, underwent normalization using the z-score method [48]. Figure 3 illustrates the above-described EEG signal processing and feature-extracting workflow.

### 2.7. Statistical and Correlation Analyses

The IBM Statistical Package for the Social Sciences (SPSS) version 28 was employed for data analysis. Descriptive analyses were conducted to describe the sample and other variables under study, along with correlation analyses between the CAIDE score, MMSE, and EEG-extracted PSD-based features. These correlations were analyzed with the study’s total sample to explore the association between the variables of interest. To test our research questions, Pearson’s correlation coefficient was used (considering a significance level set at *p* < 0.05), as all variables are treated as quantitative and do not exhibit severe deviations from normality, i.e., skewness [−3 to 3] and kurtosis [−7 to 7] [49].

For reference, in the Appendix A of this paper, we provide the individual CAIDE and MMSE scores for each participant in Table A1. Additionally, Table A2 contains the mean power values for each frequency band per channel for all subjects, along with the respective standard deviations. 

## 3. Results

To answer our research questions, Pearson’s correlation coefficient was used, as all variables are treated as quantitative and do not exhibit severe deviations from normality, i.e., skewness [−3 to 3] and kurtosis [−7 to 7] [49]. To explore the relationship between variations in risk scores assessed by the CAIDE and the characteristics of brain electrical signals recorded by EEG, as well as cognitive measures, in normative individuals, the correlation analysis was segmented into changes in low-frequency band energy separately from those occurring in high-frequency bands.

Regarding neurophysiological changes, firstly, to investigate whether the risk score positively correlates with increased low-frequency brain waves in the frontal lobe, the relationships between the CAIDE score and EEG-extracted PSD-based features for δ and θ waves were evaluated. The results showed that only the θ band had a positive and significant correlation with the CAIDE score, specifically at the mode level of FP1. In other words, higher dementia risk scores were associated with greater activation of RP(θ), with *r*(38) = 0.347, *p* = 0.033. To verify whether the participants’ ages were a factor of interference, correlations between age and the mode level of FP1 and between the CAIDE score and age were also calculated: the results showed no significance, with *r*(38) = 0.056, *p* = 0.737 and *r*(38) = 0.091, *p* = 0.585, respectively. Additionally, to verify whether the dementia risk score positively correlates with a decrease in high-frequency brain waves in the frontal lobe, the relationships between the CAIDE score and EEG-extracted PSD-based metrics for α, β, and γ waves were evaluated. However, none of them showed a correlation.

In turn, regarding cognitive performance, to ascertain whether the dementia risk score is negatively correlated with cognitive performance measures, the relationship between the CAIDE risk score and the score obtained in the MMSE was calculated. A significant negative correlation was found between these two variables, with *r*(38) = −0.369, *p* = 0.023. This means that higher dementia risk scores are associated with poorer performance on the MMSE.

Based on these results, an exploratory correlation analysis was conducted to assess whether variations in MMSE scores translate into neurophysiological changes. To determine whether poorer performance on cognitive measures is correlated with increased low-frequency brain waves in the frontal lobe, the relationships between the MMSE score and EEG-extracted features for δ and θ bands were examined. The MMSE score was found to be negatively correlated with the δ band, specifically at $RP{\left(\delta \right)}_{FP2}^{mean}$ (*r*(38) = −0.369, *p* = 0.023) and $RP{\left(\delta \right)}_{FP2}^{median}$ (*r*(38) = −0.370, *p* = 0.022). In other words, poorer performance on the MMSE was associated with greater RP(δ). The θ band did not show a correlation with cognitive measures. To investigate whether poorer performance on cognitive measures is correlated with decreased high-frequency brain waves in the frontal lobe, the relationships between the MMSE score and EEG-extracted PSD-based metrics for α, β, and γ waves were analyzed. Only the low-β band was significantly correlated, specifically at $RP{\left(\beta 1\right)}_{FP1}^{mode}$ (*r*(38) = −0.343, *p* = 0.035). This means that lower MMSE scores were associated with greater activation of the low-β band.

**Figure 4:**
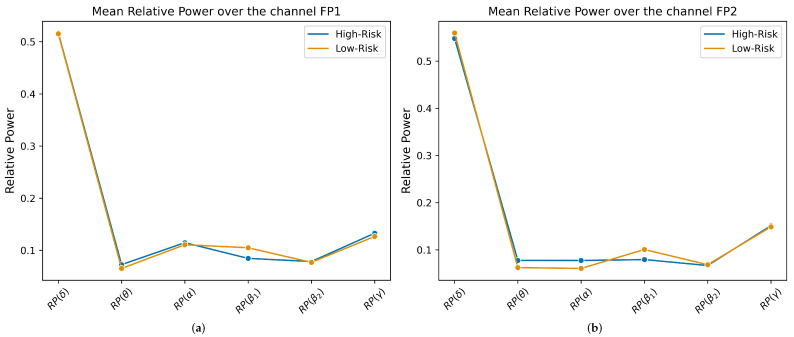
Mean multi-band relative power of groups over different channels. (**a**) FP1—mean relative power over EEG bands in different groups. (**b**) FP2—mean relative power over EEG bands in different groups.

To demonstrate the relationship between high-risk dementia and the EEG slowing down, participants were divided into two groups. The Low-Risk Group (n = 16) and the High-Risk Group (n = 22) were established based on the scores from the CAIDE scale, specifically 0–9 and 10–13, as carried out by Kivipelto et al. in their study [20]. Furthermore, a line graph was drawn for each channel, FP1 and FP2, with two risk groups: “High-Risk” in blue and “Low-Risk” in orange (see Figure 4, and for more information, consult Table A3). The presented graphs show different groups’ average relative power over EEG bands. In both channels, it is observed that there is an unmistakable energy transfer from the high-frequency bands to the low-frequency bands. Although in channel FP1, the curves for both groups are pretty similar, in channel FP2, there is a smooth difference in β1, where the Low-Risk Group exhibits slightly higher relative power, and in θ and α, where the High-Risk Group shows a slightly higher power than the Low-Risk Group. These variations in brain activity between the groups suggest spectral deceleration. This can also be corroborated by the mean Individual Alpha Frequencies (IAFs) [50,51] of both groups: (1) in FP1, the mean IAF value is 8.42 Hz ± 1.07 for the Low-Risk Group and 8.02 Hz ± 0.95 for the High-Risk Group; (2) in FP2, the mean IAF value is 8.01 Hz ± 0.92 for the Low-Risk Group and 7.63 Hz ± 0.74 for the High-Risk Group. This demonstrates that High-Risk Groups exhibit a tendency to shift to the left of the Alpha Power Spectral Peak, which has also been reported in Alzheimer’s Disease Patients [52,53,54].

## 4. Discussion

Although analyzing the transition from normal to pathological aging is essential and relevant, particularly by exploring the neurophysiology of risk factors, this knowledge is still limited. Therefore, the present study aimed to investigate whether the variation in dementia risk scores obtained from CAIDE is associated with changes in the electrical signal captured by EEG in the frontal lobe and cognitive measures in normative individuals.

Previous longitudinal studies have demonstrated an association between structural changes in the brain and CAIDE risk score [22,23]. However, limited research has investigated the neurophysiological basis underlying these factors in the pre-dementia stage in cognitively healthy individuals. Thus, this study has two main objectives: (1) investigate the relationship between variations in dementia risk scores, as assessed by CAIDE, and the characteristics of brain electrical signals recorded by EEG, as well as cognitive measures, in normative individuals; (2) explore whether a deceleration effect in the spectrum, similar to that observed in dementia, appears in individuals with high dementia risk scores before the onset of cognitive decline.

Correlational exploratory analyses were conducted. It was found that individuals with a higher CAIDE dementia risk activated the θ band more. These findings align with the literature mentioned above, indicating that in resting-state EEG measurements, high-frequency bands (i.e., α and β) shift to low-frequency bands [29]. The results are similar to those reported by Rodrigues and colleagues (2021) [39], where patients with mild cognitive impairment associated with Alzheimer’s disease, compared to healthy individuals, also exhibited a more pronounced deceleration effect in the θ wave. Increased activation of the θ band is a prominent characteristic of the deceleration effect in electroencephalographic signals present in individuals with dementia, being one of the first changes detected in the early stages of dementia [30]. Therefore, it is not surprising that the θ band stands out, as it captures information from the hippocampus (the main structure involved in memory consolidation processes), with θ frequency potential oscillations considered critical for acquiring new information [55]. In practice, increased activation of this band is associated with decreased cerebral compliance and has been reported to be related to amyloidosis. Specifically, an increase in RP(θ) in the frontal cortex has been observed in individuals with higher amyloid deposition compared to those with lower deposition [56].

Additionally, it was observed that the CAIDE dementia risk score did not correlate with a decrease in high-frequency waves. In other words, this process does not parallel the changes analogous to dementia at the same pace. This effect may be because the individuals under study have no cognitive deficits as measured by MMSE. Therefore, the risk of dementia alone should not impact neuronal activity in the same way as an established pathological condition, making it consistent that the observed changes are at a lower degree. The signal propagation effect highlights the importance of paying attention to the identification of information loss [39], as demonstrated by the IAF and the line graphs, where brain activation is reorganized, altering the frequencies of each band. Thus, following the deceleration effect documented in numerous studies with dementia populations, changes in higher- and lower-frequency waves are also visible in individuals with a higher risk of dementia. The early identification of dementia risk serves as a warning for controlling risk factors, aiding in preventing this condition. Thus, the findings reinforce the importance and relevance of using the CAIDE dementia risk score before experiencing complaints or cognitive alterations. It should be clarified that this is not a causal relationship, meaning that an individual with a high CAIDE dementia risk score does not inevitably present modifications in the electrical signal captured by the electroencephalogram and vice versa. Here, we only find a non-directional relationship between these two instruments. Nonetheless, the results help to ensure that CAIDE is an instrument that, being relatively easy to apply to the general population, can help to develop a preventive perspective in healthcare services. It was also found that a higher CAIDE risk score is associated with poorer performance in cognitive measures. These results align with the literature on risk factors, highlighting a greater likelihood of cognitive impairment related to higher levels of these factors [13]. Since CAIDE is a multifactorial risk score, it is logical that combining various factors into a global risk score is also associated with poorer performance on tests assessing cognitive domains, such as the MMSE.

Furthermore, it was observed that the worse the performance on the MMSE, the greater the RP(δ). These data are consistent with previous studies that have aimed to establish a relationship between spectral EEG measures and neuropsychological instruments, notably a significant correlation between MMSE results and increased RP(δ) and RP(θ) in individuals with dementia [57].

Contrary to expectations, it was found that lower MMSE scores were associated with greater RP(β). As mentioned earlier, the β band is generally associated with higher cognitive processes such as attention, concentration, and active mental activity [58]. One possible explanation could be the occasional presence of a compensatory mechanism in individuals with poorer cognitive efficiency as a brain response to preserve a minimum level of performance, involving increased recruitment of this band (resource allocation), akin to other compensatory mechanisms [59].

In terms of theoretical implications, this study fills a gap in the literature regarding the impact of risk factors on neuronal activity in normative individuals. In practice, it may translate into advocating for awareness campaigns to reduce risk factors, thereby improving quality of life. A study that showed promising results of intervention in individuals with a high CAIDE dementia risk was the Finnish Geriatric Intervention Study to Prevent Cognitive Impairment and Disability (FINGER), which demonstrated that after two years of a multi-domain intervention (e.g., cognitive training, nutrition, physical activity), maintenance or improvement in mental status could be observed in community-dwelling older adults aged 60 to 77 at risk of dementia [60].

However, the practical implementation period of the intervention remains largely unexplored, and it is currently impossible to identify who among individuals with “no”, “low”, or “high” CAIDE risk would benefit most from this intervention. As mentioned, it appears advantageous for individuals with high risk, but the literature is divergent, with Ngandu’s study [60] being pioneering. In future research, it is essential to understand and delineate this period so that interventions can be extended to the broader population. Therefore, it is suggested to conduct exploratory studies using an experimental protocol with the analysis of electroencephalographic signals to compare pre- and post-intervention results among individuals with “no”, “low”, or “high” CAIDE risk.

Future studies should include a more in-depth neuropsychological assessment, rather than just screening tests, to understand whether the risk factors affect any cognitive domains. Future research should also aim to include a larger and more balanced group population for both study groups (“low” and “high” CAIDE risk score groups) to ensure a more reliable generalization of the findings. Additionally, future studies are recommended to include a more heterogeneous population regarding educational attainment to capture differences in risk and its impact on EEG signals. It is worth noting that higher levels of education are considered promoters of cognitive reserve [13]. In the current investigation, most participants had zero to six years of schooling.

The choice to measure changes in EEG signals in the prefrontal channels optimized the detection process; however, it may also be considered a study limitation. Therefore, it is additionally suggested to include the application of temporal electrodes.

## 5. Conclusions

This is a highly relevant and innovative topic from a prevention perspective, helping to raise awareness in society for the monitoring and controlling of these factors as early as possible, emphasizing their impact and importance in reducing the incidence of dementia.

In summary, the findings suggest that the CAIDE score appears to identify individuals who, despite not having cognitive impairments, already exhibit brain activity similar to that found in individuals with dementia. Thus, it can be asserted that there is already a slowing effect observable in the spectrum due to losses in signal propagation, compensated by an increase in θ-band power. Furthermore, the results contribute to a measure of convergent validity between CAIDE and cognitive changes (even within normative values).

These discoveries provide insights into the relationship between EEG slowing and dementia risk, aiding in a better understanding of the underlying mechanisms of the disease. Consequently, they advocate for early monitoring and controlling risk factors to decrease incidence. However, no causal relationship has been established, necessitating further in-depth research. Dementia is a complex multifactorial condition, and the correlation between dementia risk and neurophysiological signals is just one component of this constantly evolving area of study.

## Figures and Tables

**Table 1:** Sample characterization.

Variables (N = 38)	Minimum	Maximum	Average	Standard Deviation	Asymmetry	Kurtosis
Total Cholesterol	117	268	187.11	38.68	0.276	−0.798
Systolic Hypertension	95	220	143.26	25.76	0.778	1.340
Diastolic Hypertension	49	160	77.61	17.84	2.653	11.824
BMI	19	43	28.08	4.85	0.870	1.792
MMSE	23	30	27.65	1.85	−0.764	0.114
BDI	5	19	10.38	3.26	0.665	0.421
CAIDE	4	13	9.5	2.06	−0.604	0.383

**Table 2:** CAIDE dementia risk score adaptation.

Variables	Attributed Points	Evaluation Method
Age		
<47 years	0	
47–53 years	3	Sociodemographic Questionnaire
>53 years	4	
Gender		
Female	0	Sociodemographic Questionnaire
Male	1	
Education		
≥10 years	0	
7–9 years	2	Sociodemographic Questionnaire
0–6 years	3	
Systolic Hypertension		
≤140 mmHg	0	Provided by the institution
>140 mmHg	2	
BMI		
≤30 Kg/m^2^	0	Provided by the institution
>30 Kg/m^2^	2	
Total Cholesterol		
≤200 mg/dL	0	Provided by the institution
>200 mg/dL	2	
Physical Activity *		
Active	0	Provided by the institution
Inactive	1	

* Physically Active = practices physical activity at least two times per week for 20–30 min each, with the presence of sweating and lack of air.

**Table 3:** Features extracted, corresponding equations, and short description.

Feature	Equation	Description
Relative power of δ	$RP\left(\delta \right)\;=\;\sum_{1Hz}^{4Hz}PSD_{n}\left(f\right)$	Relative power of the delta frequency band (1 Hz to 4 Hz).
Relative power of θ	$RP\left(\theta \right)\;=\;\sum_{4Hz}^{8Hz}PSD_{n}\left(f\right)$	Relative power of the theta frequency band (4 Hz to 8 Hz).
Relative power of α	$RP\left(\alpha \right)\;=\;\sum_{8Hz}^{13Hz}PSD_{n}\left(f\right)$	Relative power of the alpha frequency band (8 Hz to 13 Hz).
Relative power of β1	$RP\left(\beta 1\right)\;=\;\sum_{13Hz}^{19Hz}PSD_{n}\left(f\right)$	Relative power of the beta1 frequency band (13 Hz to 19 Hz).
Relative power of β2	$RP\left(\beta 2\right)\;=\;\sum_{19Hz}^{30Hz}PSD_{n}\left(f\right)$	Relative power of the beta2 frequency band (19 Hz to 30 Hz).
Relative power of γ	$RP\left(\gamma \right)\;=\;\sum_{30Hz}^{40Hz}PSD_{n}\left(f\right)$	Relative power of the gamma frequency band (30 Hz to 40 Hz).

## Data Availability

Data are available on request due to privacy reasons.

## Supplementary Materials

The following supporting information can be downloaded at: https://www.mdpi.com/article/10.3390/brainsci14111120/s1.

## Appendix A

### Appendix A.1

**Table A1:** Participants’ individual CAIDE and MMSE scores.

Subject	MMSE	CAIDE
1	30	6
2	28	9
3	27	10
4	25	9
5	27	10
6	27	7
7	27	10
9	26	11
11	29	8
13	28	11
14	30	8
16	30	12
17	29	10
18	23	12
21	29	12
22	27	11
24	24	12
26	27	10
27	28	10
28	30	10
29	30	10
30	29	8
31	28	8
32	29	8
33	28	11
34	30	13
35	27	11
36	30	10
37	28	10
38	29	9
39	30	7
40	27	13
41	25	9
42	26	8
43	29	8
44	26	11
46	30	5
47	30	4

### Appendix A.2

**Table A2:** Average power values with standard deviations across the different EEG frequency bands per channel.

Frequency Band Relative Power	Average ± Standard Deviation	
	FP1	FP2
RP(δ)	0.503 ± 0.018	0.510 ± 0.019
RP(θ)	0.071 ± 0.036	0.075 ± 0.037
RP(α)	0.118 ± 0.052	0.116 ± 0.046
RP(β1)	0.090 ± 0.032	0.085 ± 0.042
RP(β2)	0.079 ± 0.022	0.061 ± 0.014
RP(γ)	0.139 ± 0.062	0.152 ± 0.063

### Appendix A.3

**Table A3:** Average power values with standard deviations across the different EEG frequency bands per channel per group.

Frequency Band Relative Power	Group	Average ± Standard Deviation	
		FP1	FP2
RP(δ)	Low-Risk	0.500 ± 0.019	0.511 ± 0.018
	High-Risk	0.504 ± 0.018	0.510 ± 0.020
RP(θ)	Low-Risk	0.068 ± 0.037	0.066 ± 0.033
	High-Risk	0.074 ± 0.036	0.083 ± 0.038
RP(α)	Low-Risk	0.116 ± 0.051	0.116 ± 0.051
	High-Risk	0.119 ± 0.053	0.116 ± 0.044
RP(β1)	Low-Risk	0.099 ± 0.033	0.092 ± 0.050
	High-Risk	0.083 ± 0.030	0.080 ± 0.036
RP(β2)	Low-Risk	0.078 ± 0.019	0.063 ± 0.016
	High-Risk	0.080 ± 0.024	0.060 ± 0.014
RP(γ)	Low-Risk	0.139 ± 0.064	0.153 ± 0.074
	High-Risk	0.140 ± 0.063	0.151 ± 0.056
