# Supplementary material for: Exploring the Relationship Between CAIDE Dementia Risk and EEG Signal Activity in a Healthy Population
Source: Brain Sci. 2024 Nov 4;14(11):1120. doi: 10.3390/brainsci14111120 (PMC11592169; doi:10.3390/brainsci14111120)
Supplement: Supplementary file 1 [file brainsci-14-01120-s001.zip › brainsci-3278047-supplementary.pdf]

CATOLICA
FACULTY OF HEALTH SCIENCES
AND NURSING
LISBOA·PORTO

Principal Investigator:
Alice Rodrigues Manuel
s-alromanuel@ucp.pt
Portuguese Catholic University, Lisbon

Supplemental File S1:

INFORMED CONSENT

Project Title: ***"Preliminary Study: Correlation between Dementia Risk and EEG Signal Deceleration Effect"***

This document invites you to voluntarily participate in a study as part of the Master's dissertation project in Neuropsychology at Portuguese Catholic University. Please read the entire contents of this document carefully. Do not hesitate to ask the principal investigator for more information if you are not completely clear.

1) The **aim of the study** is to investigate whether there is an association between the variables "Risk of Dementia" and "Changes in the Electrical Signal Captured by Electroencephalogram (EEG)."

2) Your participation involves verbally answering some questionnaires (paper test) that will be presented to you, including:

- ***Sociodemographic Data Questionnaire*** **-** Consisting of questions related to the sociodemographic characteristics of each participant, such as gender, age, education, physical activity, professional occupation, etc.
- ***Mini Mental State Examination*** **-** A screening instrument for global cognitive status, assessing various domains of cognition.
- ***Beck Depression Inventory*** **-** A self-assessment questionnaire for depression, where participants are asked to respond about the presence of certain symptoms over the past two weeks.

- ***Pittsburgh Sleep Quality Index* -** Participants are asked to respond as accurately as possible about their sleep habits over the past month.
- ***Montreal Cognitive Assessment* -** A screening instrument for various cognitive functions (attention, memory, calculation, etc.) to characterize the cognitive status of the participants.

Additionally, your dementia risk level will be calculated using the CAIDE Dementia Risk Score, which authorizes the institution to provide your blood pressure, body mass index, and cholesterol records. Finally, your electroencephalographic records (Resting EEG) will also be obtained – you may experience some discomfort due to the conductive gel that will be applied to your scalp.

(The mentioned instruments do not have a diagnostic purpose)

3) **Duration:** Your participation will take place in two sessions, each with an average duration of 40 minutes.

4) **Benefits and Risks:** Your participation in the study will contribute to the advancement of scientific knowledge in the fields of neuroscience and aging, particularly regarding the impact that risk factors for dementia may have on changes in the electrical signals resulting from neuronal communication, as captured by the electroencephalogram. However, there are no direct benefits associated with your participation in this study. Also, no risks to your physical or psychological well-being are anticipated.

5) **Data Collection:** The data collected will be processed subsequently using computerized methods for validation and analysis. The data will be used exclusively for this study and for academic purposes, after which it will be destroyed.

6) **Confidentiality:** The only person with access to the information provided will be the principal investigator. Your personal data will not be disclosed at any time and will be kept confidential.

7) **Voluntary Participation:** Your participation is entirely voluntary. Even after the study has commenced, you may withdraw at any time without penalty. You need only inform the principal investigator of your decision to withdraw.

8) **Responsible Supervisors:** Professor Maria Vânia Nunes, mnunes@ucp.pt – Portuguese Catholic University, Lisbon, and Professor Pedro Miguel Rodrigues, pmrodrigues@ucp.pt.

9) **Data Protection Officer:** Dr. Frederica Campos de Carvalho, compliance.rgpd@ucp.pt, 217214179, Portuguese Catholic University, Lisbon.

I declare that I have read this document and that I am participating voluntarily in the study. I have had the opportunity to ask questions, and any doubts have been clarified.

Date: __ / __ / ____

Principal Investigator:

Date: __ / __ / ____

Sociodemographic Data Questionnaire

Date: ____/____/_____ **Coding:**______

Age: ____________________

Gender: Female______ Male______

Years of Education:

>10 years ____

7-9 years ____

0-6 years ____

Profession:________________________

Family history of dementia: yes ______ No______

(A family history of dementia is considered positive if at least one parent has been clinically diagnosed with dementia.)

Physical Activity:

How often do you engage in physical activity that lasts at least 20 to 30 minutes and causes shortness of breath or sweating?

1)____5 times per week or more frequently

2)____4 times per week

3)____3 times per week

4)____2 times per week

5)____once a week

6)____less than once a week

7)____I have a disability or illness that prevents me from exercising

(Physical inactivity is defined as a frequency of less than 2 times per week)

Do you take medication? Yes___ No___ _____________________________________
